# Metabolic Reprogramming in Thyroid Carcinoma

**DOI:** 10.3389/fonc.2018.00082

**Published:** 2018-03-23

**Authors:** Raquel Guimaraes Coelho, Rodrigo S. Fortunato, Denise P. Carvalho

**Affiliations:** ^1^Laboratório de Fisiologia Endócrina, Instituto de Biofísica Carlos Chagas Filho, Universidade Federal do Rio de Janeiro, Rio de Janeiro, Brazil; ^2^Laboratório de Radiobiologia Molecular, Instituto de Biofísica Carlos Chagas Filho, Universidade Federal do Rio de Janeiro, Rio de Janeiro, Brazil

**Keywords:** glycolysis, glutaminolysis, Warburg effect, thyroid cancer, hypoxia-inducible factor, hexokinase, AMP kinase, mammalian target of rapamycin protein

## Abstract

Among all the adaptations of cancer cells, their ability to change metabolism from the oxidative to the glycolytic phenotype is a hallmark called the Warburg effect. Studies on tumor metabolism show that improved glycolysis and glutaminolysis are necessary to maintain rapid cell proliferation, tumor progression, and resistance to cell death. Thyroid neoplasms are common endocrine tumors that are more prevalent in women and elderly individuals. The incidence of thyroid cancer has increased in the Past decades, and recent findings describing the metabolic profiles of thyroid tumors have emerged. Currently, several drugs are in development or clinical trials that target the altered metabolic pathways of tumors are undergoing. We present a review of the metabolic reprogramming in cancerous thyroid tissues with a focus on the factors that promote enhanced glycolysis and the possible identification of promising metabolic targets in thyroid cancer.

## Introduction

Thyroid cancers are the most common endocrine tumors and are more prevalent in women and elderly individuals (1, 2). Although the incidence of thyroid tumors can be high in a population (1), epidemiological studies indicate that only a small fraction of tumors are malignant (1, 3). Some rare thyroid malignancies that derive from the follicular thyroid epithelia are poorly differentiated and frequently metastasize early (4). In contrast, differentiated thyroid carcinomas (DTCs) generally exhibit a good prognosis and excellent outcomes (3, 5, 6).

The therapy for intermediate and high-risk DTCs, includes a combination of surgery, radioiodine ablation, and thyroid stimulating hormone (TSH) suppressive therapy. However, although DTCs are slow-growing tumors, disease recurrence can occur (4–6). In approximately 10% of DTC recurrence cases, tumor progression leads to a more aggressive phenotype, metastatic spread, and further loss of iodide uptake ability (4–6).

In the past several years, targeted therapeutic approaches have been developed as an option to control disease progression. Unfortunately, multikinase inhibitors that target angiogenesis and oncogenic pathways have deleterious side effects and do not result in a cure. Although a significant increase in the progression-free survival rate has been observed with the use of multikinase inhibitors, the diversity of tumor types, and tumor resistance that develops during progression impede this unique therapeutic strategy (5, 6). However, tumor metabolic behavior is known to become quite different as cells to transform into malignant cells. Interestingly, some metabolic feature changes are observed in several tumor types. Although the physiological function of the thyroid gland is very well described, its metabolic control and adaptations remain elusive, especially in thyroid cancer. In this review, we discuss some metabolic adaptations identified in thyroid carcinoma that could be used as future therapeutic targets in this disease.

## Main Molecular Events Related to Thyroid Carcinogenesis

The incidence of thyroid cancer has increased in many countries compared to that of other human cancers (7). Approximately 90% of non-medullary thyroid malignancies that originate from thyroid follicular cells are classified as well-DTCs. DTCs are subdivided into follicular thyroid carcinoma (FTC) and papillary thyroid carcinoma (PTC), the latter of which is more prevalent, accounting for approximately 80% of DTCs (8, 9). The oncocytic or Hurthle cell tumors represent approximately 3–5% of follicular thyroid neoplasms (10), and they may be benign (variant of follicular adenoma) or malignant (variant of follicular carcinoma and variant of papillary carcinoma). The main characteristic of Hurthle cell carcinomas is the presence of at least 75% large oxyphilic cells that are characterized by abundant mitochondria (11), and previous exposure to radiation might be a risk factor for the development of Hurthle cell carcinoma and some subtypes of papillary thyroid cancers (8–16).

Undifferentiated thyroid carcinomas represent less than 5% of thyroid malignancies and are frequently associated with disease recurrence and death (4, 8, 9). Finally, medullary thyroid carcinoma, which derives from parafollicular C cells, produces calcitonin and accounts for approximately 5% of thyroid carcinomas (9).

The different morphologic subtypes of DTC are due to specific genetic alterations. RAS is a proto-oncogene that encodes a family of GTPases that are activated through tyrosine kinase receptor pathways involved in the regulation of cell differentiation and proliferation. RAS mutations can be found in 20–25% of all human tumors and in up to 90% of pancreatic cancers (17). Regarding thyroid cancer, RAS mutations are found in approximately 10% of thyroid cancer cases, mainly the follicular variant (18–20). Mutations of the proto-oncogene RAS induce changes in Ras protein, leading to its constitutive activation inside the cell. Although the prevalence of RAS mutations in the thyroid is low, they are associated with aggressive behavior in several other types of cancer (21–23).

Another human gene involved in thyroid carcinogenesis is BRAF. The B-Raf protein is a serine/threonine kinase that is activated downstream of Ras and is involved in cell growth control (24). Mutations in B-Raf induce its constitutive activation, subsequently activating the downstream mitogen-activated protein kinase (MAPK) signaling pathway (20, 24). Although other mutations have been described, BRAF^V600E^ (the substitution of valine for glutamic acid in residue 600) is the most frequent mutation (24). In PTC, BRAF is the predominant mutation (30–40%) and is considered an initiating event in papillary thyroid carcinogenesis (18, 25–27).

Genetic alterations in the RET gene have also been found in several types of cancers (28). RET encodes a transmembrane protein receptor with an intracellular portion containing a tyrosine kinase that triggers its autophosphorylation, initiating intracellular signaling related to the stimulation of the RAS/ERK and PI3 kinase/AKT cascades (25, 28, 29). In addition to BRAF, RET mutations are also responsible for thyroid cancers (9, 16, 25). Somatic point mutations in RET are associated with familiar or sporadic medullary thyroid cancer, since RET is normally expressed in C cells, but not in follicular thyroid cells (29). In PTC, RET translocations (RET/PTC) can be identified in approximately 20% of the cases (9).

In some tumors, PAX-8, which encodes a transcription factor associated with thyroid development, has been implicated in carcinogenesis (30). Tacha et al. (30) found that mutated PAX8 is expressed in some follicular thyroid cancers due to somatic rearrangement leading to the fusion of PAX-8 with PPARγ1 (peroxisome proliferator-activated receptor gamma 1) (30). In FTC, the frequency of PAX8/PPARγ1 rearrangement is estimated to be approximately 30%, but this is not observed in PTCs. RAS mutations are also found in FTC (9, 20, 21).

The therapeutic approach for thyroid cancer may depend not only on the tumor initial mutational status, which leads to different cell biology characteristics, but also to hallmarks related to tumor progression. Some of these molecular changes result in specific metabolic alterations that might contribute to metastasis and a worst prognosis.

## Cancer Cell Metabolism

**Figure 1:**
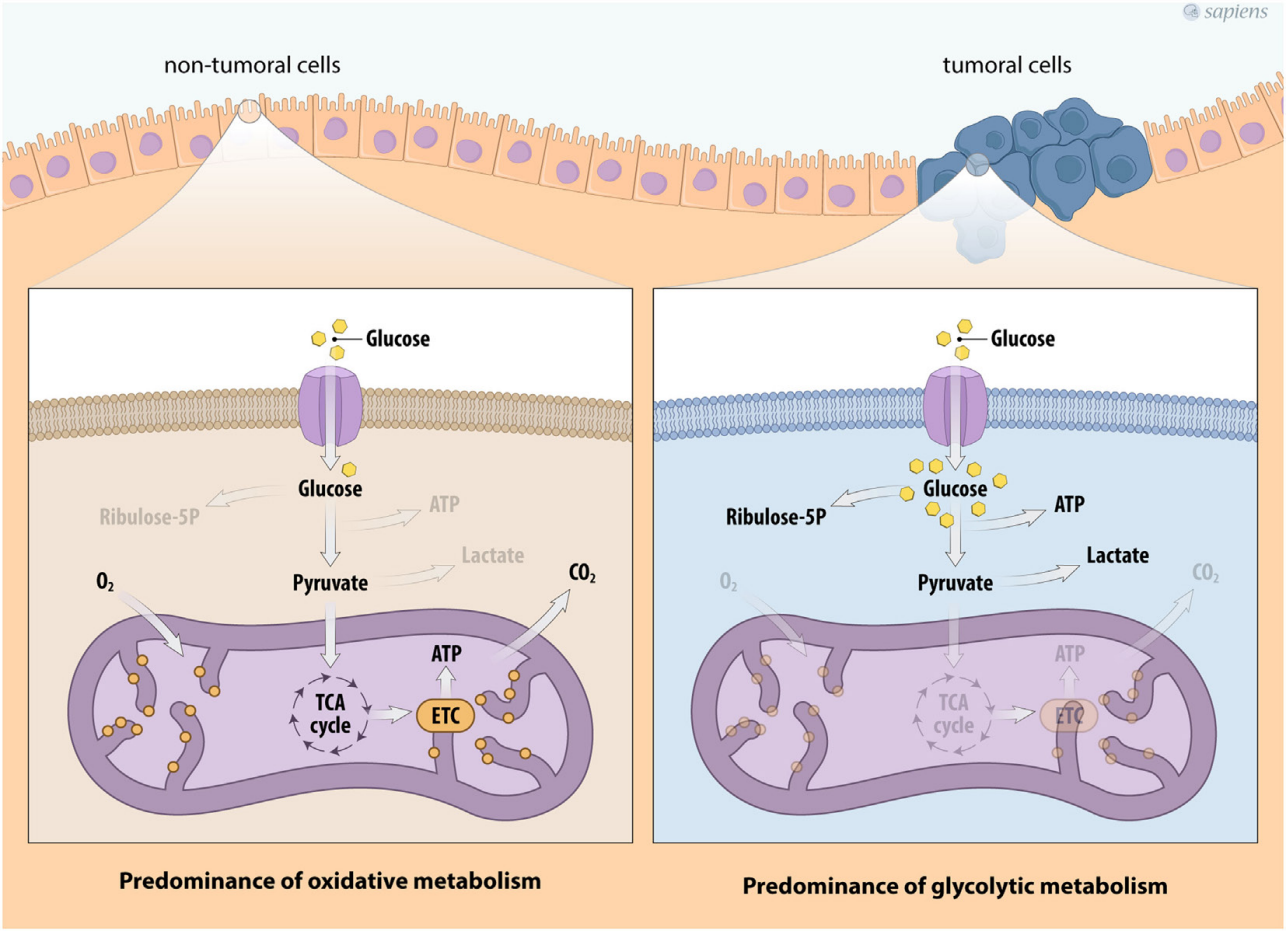
Metabolic profile of cancer cells. Schema describing the metabolic reprogramming of tumor cells with increased glucose uptake and glycolysis that is uncoupled from oxidative phosphorylation. Cancer cell metabolism is characterized by enhanced glycolysis and the phosphate pentose pathway. This aerobic glycolytic phenotype, however, confers the generation of high amounts of lactate. Abbreviations: TCA, tricarboxylic acid; ETC, electron transport chain.

In the past several years, there has been significant interest in the metabolic reprogramming of cancer cells. In general, non-tumor cells use energy substrates, such as glucose and fatty acids, to generate energy under aerobic conditions. Glucose metabolism is initiated by glycolysis, the pathway that converts one glucose molecule into two molecules of pyruvate, which are transported to the mitochondria for oxidation. The glycolytic pathway is generally coupled with the mitochondrial tricarboxylic acid (TCA) cycle due to the action of the pyruvate dehydrogenase (PDH) protein complex that converts pyruvate into acetyl-CoA. The TCA cycle consists of successive reactions that lead to the transfer of electrons to NAD^+^/FAD^+^ for the generation of NADH/FADH_2_, forming a wide range of metabolic intermediaries that are involved in various biosynthetic routes. The TCA cycle allows electron transfer to oxygen and generates a proton gradient across the inner mitochondrial membrane that is necessary for ATP synthesis in a process called oxidative phosphorylation (OXPHOS), a metabolic strategy that enables the cellular production of a greater amount of ATP (Figure 1).

Under physiological stress conditions, such as hypoxia or rapid intracellular ATP decreases, the cell increases its anaerobic metabolism, producing higher amounts of lactate from pyruvate. Interestingly, most cancer cells show a constitutive stress metabolic phenotype due to their high proliferation rates, which induces an elevated ATP demand compared to non-tumor cells. Therefore, cancer cells show variable energy substrate selection and a metabolic shift occurs to maintain cell proliferation and survival (31–35). Figure 1 summarizes the major metabolic modulation on energy flux in tumor cells.

Decades ago, Otto Warburg described the first tumor-specific metabolic characteristic, the so-called Warburg effect or aerobic glycolysis, which is considered the key metabolic hallmark of cancer (31). The Warburg effect is an alteration of cellular metabolism in which the glycolysis pathway is upregulated even in the presence of normal or high oxygen tension, resulting in the phenomenon of aerobic glycolysis. However, such metabolic reprogramming in cancer cells represents an energy compensation strategy, since the efficiency of ATP production by glycolysis is much lower than that by OXPHOS (36). Part of this strategy is the upregulation of plasma membrane glucose transporters (GLUTs), a feature that is relatively common in many tumor types and is easily identified by non-invasive imaging positron emission tomography (PET) using fluorodeoxyglucose (34–37). In addition to increased glucose uptake, changes in key enzymes involved in glucose utilization can also be observed (34, 35). Some tumors show increased expression and activity levels of hexokinase (HK) isoforms, phosphofructokinase (PFK1), 6-phophofructo-2-kinase/fructose-2,6-biphosphatase (PFK2), aldolase (ADO), phosphoglycerate kinase (PGK), enolase (ENO), and pyruvate kinase (PK) (37). All these changes can increase pyruvate production from glucose breakdown.

The higher glycolytic flux observed in cancer cells, is not accompanied by increased rates of pyruvate oxidation, but lactate fermentation seems to be higher (Figure 1). Although this phenomenon is not fully understood, in recent years, significant progress has been made regarding the underlying molecular mechanisms related to neoplastic transformation and the Warburg effect (32–39). First, the lactate dehydrogenase enzyme (LDH) consumes part of the pyruvate formed by glycolysis to regenerate NAD^+^ from the NADH produced by glucose breakdown, allowing a higher rate of glycolysis. Second, the LDH is a reversible enzyme that can generate NADH and pyruvate, thus contributing to mitochondrial OXPHOS. Third, both lactate and pyruvate can be transported from the cytosol to the mitochondria, or they can be secreted out of the cell. Lactate transport is mainly executed by monocarboxylate transporters (MCTs), a family of more than 14 types of transporters (38). Secreted lactate and pyruvate can be taken up by adjacent cancer cells and provide a feedforward mechanism for tumor growth, a phenomenon that is called as the reverse Warburg effect (39–41).

In adverse conditions, such as fluctuating oxygen tension, which is observed in solid tumors in the setting of poor blood vessel irrigation, glycolysis allows cancer cells to live in hypoxic conditions. However, survival at a lower oxygen tension has consequences, such as excessive lactate production and decreased extracellular pH, which leads to a microenvironment that favors the extrusion of tumor cells from primary tissues (33, 36, 40, 41). Therefore, aerobic glycolysis can generate lactate, an important metabolite that favors tumor invasion and progression, which is advantageous for proliferating cells (33, 35, 36, 39). Consequently, the idea that the Warburg effect is due to mitochondrial dysfunction has changed. In many tumor models, OXPHOS changes are important to maintain growth and progression, indicating that OXPHOS may be an important metabolic target in cancer treatment (42–46).

**Figure 2:**
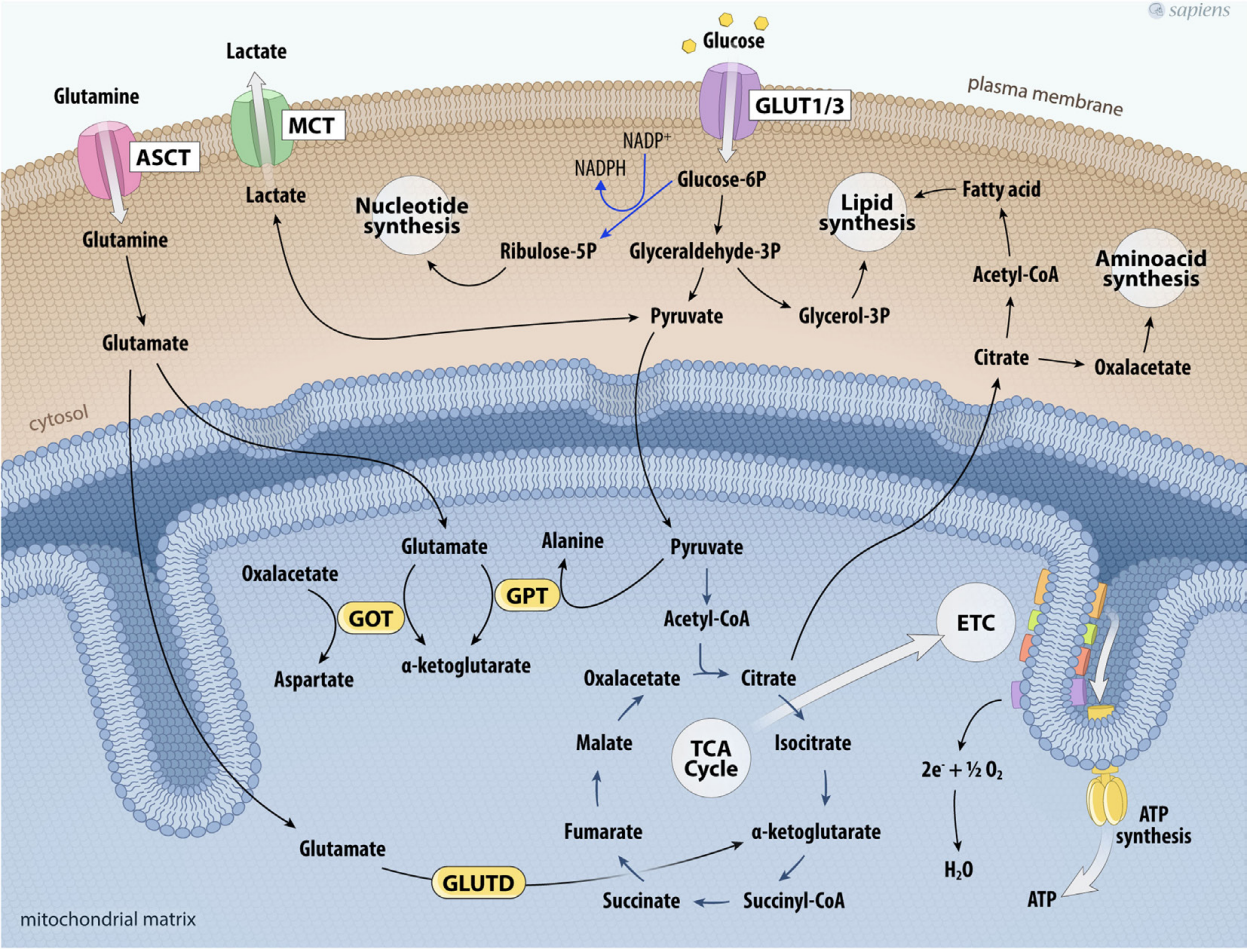
Glutaminolysis and glucose metabolism in cancer cells. The higher glycolytic pathway contributes not only to the production of ATP per glucose consumed, but also feeds other biosynthetic pathways. Deviation of glyceraldehyde-3P to glycerol-3P is important for lipogenesis. Glucose-6P can also shift toward the phosphate pentose pathway that provides ribulose-5-phosphate and NADPH to nucleotide synthesis. On the other hand, glutamine uptake maintains the anaplerotic process in the TCA cycle. Glutamine is taken up *via* the transporter ASCT and is converted into glutamate. Glutamate together with pyruvate can be metabolized by GPT producing α-ketoglutarate and alanine; glutamate is metabolized producing α-ketoglutarate and aspartate by GOT; or glutamate is metabolized by glutamate dehydrogenase (GLUTD) forming α-ketoglutarate. All these reactions contribute to support the TCA cycle. Citrate outside mitochondria contributes to the *de novo* formation of fatty acids and aminoacids. Cancer cell metabolism is also characterized by the upregulation of lactate dehydrogenase to facilitate the conversion of pyruvate to lactate, which is then secreted to the tumor microenvironment *via* the MCT. Abbreviations: ASCT, Asc-type amino acid transporter; ETC, electron transport chain; GLUT1/3, glucose transporter 1 or 3; TCA, tricarboxylic acid; GOT, glutamate-oxaloacetate transaminase; GPT, glutamate–pyruvate transaminase; MCT, monocarboxylate transporter.

During the process of tumor metabolic reprogramming, many cancer cells show greater glutamine dependence for their survival and proliferation (47–49). The elevated consumption of glutamine, a non-essential amino acid, has been documented in some tumors by assays that evaluate the uptake of two radionuclides, ^18^F or ^11^C (49). High ^18^F-glutamine uptake was related to increased sodium-dependent neutral amino acid transporter type 2 (SLA1A5) expression and by upregulated glutaminase (GLS) in several tumor models (47–49). GLS initiates glutaminolysis by converting glutamine to glutamate. The destination of glutamate depends on divergent routes. Interestingly, this pathway is involved in the maintenance of the TCA cycle and anabolic processes through the synthesis of non-essential amino acids through transamination, nucleotides (purines and pyrimidines), and fatty acids. Glutamate formed in the cytosol is transported into mitochondria, where it can be converted into α-ketoglutarate by distinct reactions catalyzed by: (a) glutamate–pyruvate transaminase, producing alanine and α-ketoglutarate; (b) glutamate–oxaloacetate transaminase (GOT), which transfers the amino group from glutamate to oxaloacetate producing aspartate and α-ketoglutarate; and finally (c) glutamate dehydrogenase (GLUTD). Together, these reactions represent the major anaplerotic pathways for the synthesis of TCA cycle intermediaries secondary to glutamine metabolism. Glutamine consumption allows the cyclic resynthesis of citrate, which is directed to the formation of fatty acids or the synthesis of amino acids (Figure 2). Moreover, glutamine metabolism participates in the generation of antioxidant agents and can also act in cell signaling (47, 49).

All these alterations of glucose and glutamine metabolism observed in cancer cells are synergic. The high glucose uptake linked to energy generation and lactate production reduces oxygen consumption. Furthermore, mitochondrial function is maintained by glutaminolysis and can support biosynthetic processes. Several studies have provided evidence that oncogenic alterations in cancer cells reprogrammed glucose and glutamine metabolism, leading to energy stress that sustains anabolic processes, which are crucial to cancer cell proliferation and progression (31–36, 40, 41, 44, 47, 49).

## Thyroid Cancer and Metabolism

Extensive documentation is available describing TSH as the main regulator of the function, proliferation, and metabolism of normal thyroid follicular cells, and well-differentiated thyroid cancer (50–56). In thyrocytes, the signaling network of TSH involves intermediates, such as protein kinase A, protein kinase C (PKC), phosphatidylinositol 3-kinase (PI3K), and MAPK. TSH activation increases glucose metabolism and oxygen consumption to support iodide transport and thyroid hormone (T3 and T4) synthesis (50–54).

Despite the importance of aerobic glycolysis, it is estimated that the ATP content produced by normal thyroid cells is mainly derived from mitochondrial respiration with low glucose consumption (55, 56). Moreover, Mulvey et al. (56) showed that glycolysis seems to be more important to sustain the pentose phosphate pathway (PPP) than ATP production in thyroid cells. The deviation of glycolysis to the PPP in the thyroid could be important to maintain the balance of NADH/NADPH generated, which is crucial for thyroid hormone synthesis.

Regarding thyroid tumors and cellular metabolism, a major aspect is the effect of oncogenes on cell metabolic shift (32). Mutated RAS induces constitutive PI3K/AKT pathway activation independently of TSH stimulation (21, 57). In many tumors, the constitutive PI3K activation results in increased glycolysis flux (58, 59), and the PI3K/AKT pathway is crucial to translocate GLUT1 from the cytoplasm to the plasma membrane in thyroid cells (53). Recently, significant increases in glycolysis, the PPP, glutamine metabolism, and the phosphoserine biosynthetic pathway were identified in colorectal cancers with the KRAS point mutation compared to wild-type cells (59).

Guo et al. (23) showed the impact of RAS mutations on the oxidative profile, which can lead to autophagy induction *in vitro* and *in vivo* in tumors. The autophagy process is characterized by catabolic cellular self-degradation in response to periods of nutrient limitations through macromolecular intracellular recycling (60). According to Guo et al. (23), in addition to providing energy substrates, the autophagy process also preserves the mitochondrial function required for cell growth, especially in models of aggressive cancers. Several years ago, it was demonstrated that in TRβ PV/PV mice, which spontaneously develop well-differentiated FTC, synergism between the KRAS^G12D^ mutation and TRβ PV occurs, leading to MYC oncogene activation and the development of the UTC phenotype (61). Interestingly, a prior study showed that in 40% of all human cancers, deregulated MYC expression could be involved in metabolic reprogramming (62). This gene encodes the Myc transcription factor (c-Myc), a multifunctional protein that plays a role in cell-cycle progression, apoptosis, and cellular transformation (62–64). Recently, Qu et al. (64) showed that BRAF^V600E^ signaling also increases c-Myc expression in the human PTC cell lineage.

**Figure 3:**
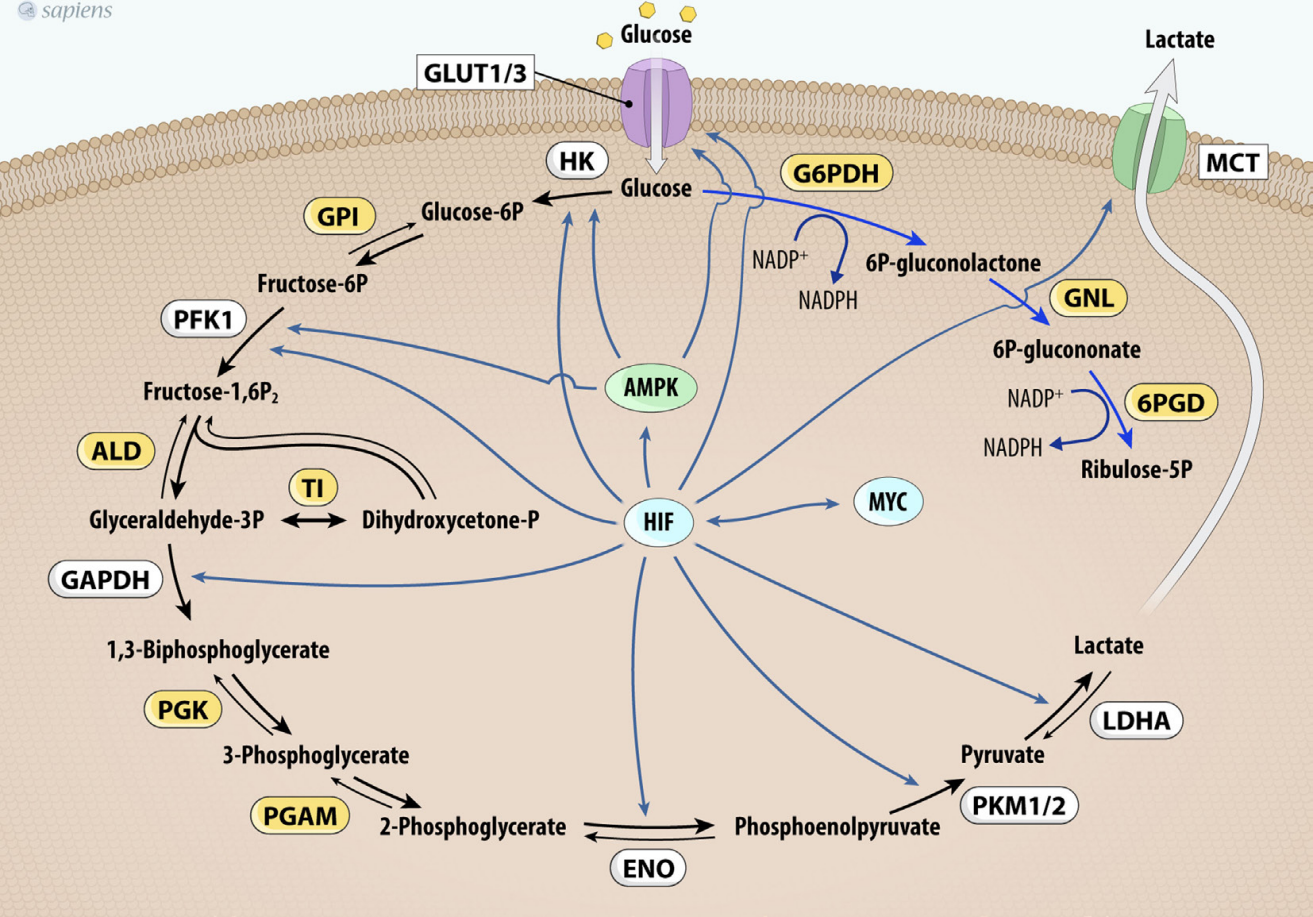
MYC and HIF-1 regulate glucose metabolism. MYC and HIF-1 are described as important regulators of key genes (in white) involved in glucose uptake and glycolysis pathway control. Abbrevations: Glut1/3, glucose transporter 1 or 3; HK, hexokinase; GPI, glucose phosphate isomerase; PFK-1, phosphofructokinase 1; ALD, aldolase; TI, triose phosphate isomerase; GAPDH, glyceraldehyde 3-phosphate dehydrogenase; PGK, phosphoglycerate kinase; PGAM, phosphoglycerate mutase; ENO, enolase; PKM1/2, pyruvate kinase isoforms 1 and 2; LDHA, lactate dehydrogenase A; G6PDH, glucose 6-phosphate dehydrogenase; GNL, gluconolactonase; 6PDG, 6-phosphogluconate dehydrogenase; MCT1, monocarboxylate transporter-1; HIF-1, hypoxia-inducible factor-1.

In addition to thyroid cancer, c-Myc overexpression has been identified in various cancers (62–64) and it upregulates the expression of genes involved in glucose metabolism (Figure 3). The first link found between c-Myc and glycolysis was the positive regulation of lactate dehydrogenase A (LDHA), the enzyme that converts pyruvate from glycolysis to lactate (65). Subsequently, GLUT-1, HK2, PFKM, and ENO1 were also identified as c-MYC targets (66–69).

Furthermore, mutated c-MYC also increases the anaerobic status of tumors, probably due to higher glycolytic flux and downregulation of OXPHOS (62, 69, 70). Pyruvate dehydrogenase kinase (PDK) is the main regulatory enzyme of mitochondrial pyruvate consumption. This protein can phosphorylate and inactivate PDH, blocking the conversion of pyruvate into acetyl-CoA and its subsequent oxidation in the TCA cycle. In hypoxic conditions, a decrease in oxidative metabolism occurs due to the inhibition of PDH by PDK. This process is modulated by hypoxia-inducible factor-1 (HIF-1), a transcription factor that promotes many cell changes in response to oxygen deprivation (66). Interestingly, cancers that harbor mutated c-Myc also increase the activity of PDK under normoxic conditions (62, 66, 69). Together, these effects contribute to increased glycolysis dependence and the development of the Warburg effect (64, 66–70). Figure 3 summarizes HIF and MYC targets on glycolytic metabolism.

The ability of tumor cells to maintain growth under hypoxic conditions is crucial to tumor progression, and the crosstalk between HIF-1 and c-Myc has been well documented (63, 66, 69). As a tumor grows, cells that can shift their metabolism in response to differences in blood supply are selected. Low oxygen tension increases HIF-1 expression and stabilization (70). Furthermore, HIF-1 is also activated by inflammatory processes, energy deprivation, and oxidative stress (71–75). Acting together, HIF-1 and c-Myc regulate several adaptations to hypoxic environments (70). As one compelling concept of the Warburg phenotype, HIF-1 enhances glycolysis not only by increasing the transcription of all glycolytic enzymes, but also by increasing their affinity for substrates (73). Moreover, HIF-1 also increases glucose transporter expression and stimulates the inhibitors of mitochondrial metabolism (71–74). In PTC, MTC, and FTC, HIF-1 expression level has been associated with a poor prognosis and metastasis (74).

The upregulation of GLUT1, HK1, HK2, PFK1, PFK2, ENO, PKM2, LDHA, and MCTs is highlighted as the most important action of HIF-1 to increase glycolysis (72, 74–76). Furthermore, HIF-1 also inhibits the PDH complex through PDK 1 overexpression, compromising the synthesis of electron donors (NADH and FADH_2_) for the respiratory chain complex and promoting the accumulation of TCA cycle intermediates (77). Therefore, HIF-1 impairs OXPHOS, reinforcing the Warburg effect (74–77). Finally, HIF-1 also cooperates with the c-Myc oncogene by reducing mitochondrial biogenesis (77, 78). For this reason, HIF-1 has been described as a central agent that promotes metabolic reprogramming in many cancer cells (73, 75–78).

In addition to its metabolic effects, HIF-1 also stimulates the formation of new blood vessels, a process called angiogenesis (70–74). The steps of tumor angiogenesis induced by HIF-1 are distinct. One possible explanation for tumor HIF-1 overexpression is the loss of von Hippel–Lindau protein activity, which is a tumor-suppressor ubiquitin ligase complex responsible for HIF-1 proteasome degradation (70, 73). In this case, HIF-1 would be able to stimulate anaerobic metabolism even under conditions with minimal fluctuations in oxygen or under normoxia (70). Gatenby and Gilles (34) suggested that the blood vessels recruited to the tumor microenvironment are disorganized and may not result in efficient cell delivery of oxygen. HIF-1 stimulates angiogenesis predominantly through the increased expression of vascular endothelial growth factor (VEGF). VEGF recruits new microvessels that allow the delivery of nutrients and expansion of the tumor mass. Some previous studies have indicated that VEGF as a risk factor of developing PTC or tumor progression (79, 80).

## Metabolic Markers in Thyroid Cancer

### GLUT1

The molecular mechanisms related to the upregulation of glucose metabolism in thyroid cancer are not yet completely understood. It has been demonstrated that ^18^F-FDG uptake can be stimulated by TSH in thyroid cancer tissue *in vivo* (81–84). However, some works have shown that, depending on the thyroid cancer subtype, ^18^F-FDG uptake can increase through a TSH-independent pathway (85, 86). In a non-tumor thyroid cell model, the action of TSH includes the activation of adenylate cyclase and PI3K (50–55). In the presence of RAS mutations, PI3K is constitutively activated, which may be partially responsible for the increased glucose uptake (57). Haber et al. (86) analyzed GLUT1 protein expression in 38 benign thyroid lesions, including follicular adenomas, Hurthle cell adenomas, nodular goiters, Hashimoto’s thyroiditis, Graves’ disease, and 28 cases of papillary, follicular, Hurthle cell, anaplastic, and medullary thyroid cancers. The authors showed that GLUT expression is frequently upregulated in thyroid cancers, but it is weakly expressed in benign nodules and in normal thyroid tissue. In addition, the localization of GLUT1 among thyroid cancers shows distinct patterns: (a) a circumferential plasma membrane focally present within the tumor in papillary carcinomas, (b) asymmetric distribution in the basilar membrane of tumor cells adjacent to the stroma and capillary blood supply, or (c) focally in the center of a tumor in metastatic or anaplastic carcinomas. Therefore, the degree and the localization of GLUT1 expression in thyroid cancers may have prognostic significance. Matsuzu et al. (87) studied the differential expression of GLUT genes in normal and pathologic thyroid tissues and demonstrated that the mRNA expression of GLUT 1, 3, 4, and 10 was evident in all thyroid tissues, but no differences were found between normal tissues and those from benign diseases. Recently, we demonstrated that GLUT1 is the predominant glucose transporter expressed in two non-tumor cell lines, PCCL3 (rat origin) and NTHY-ori (human origin) (88). In addition, the PTC cell line showed higher GLUT1 mRNA levels and protein expression compared to non-tumor cells, which may contribute to the elevated glucose uptake found in these cells (88). Recently, Naham et al. (89) analyzed 566 thyroid cancers, including PTC BRAF^V600E^ and ATC, and showed that PTC not only exhibited higher GLUT1 expression, but also higher GLUT3 expression. Moreover, the highest levels of GLUT1 expression were found in ATC, indicating that GLUT expression levels may be related to tumor aggressiveness.

### Hexokinase

In addition to the expression of GLUTs, HK expression and activity are altered in many cancers, contributing to the increased glycolytic flux (32–34, 88–92). HK catalyzes the first irreversible reaction of glycolysis and its enzymatic product is glucose-6-phosphate (G6P), which is a substrate for glycolysis or the PPP. Although there are four HK isoforms, the isoforms HK1 and HK2 seem to be overexpressed in cancer cells (92, 93). Some biochemical characteristics of HK2 are advantageous to cancer cells. First, HK2 does not have a negative regulatory site, which allows greater activity (94). Second, HK2 can bind to outer mitochondrial membrane porins and voltage-gated anion channels (VDAC), facilitating its access to newly synthesized ATP and decreasing the negative feedback of G6P for glucose phosphorylation (94–98). Third, the binding of HK2 to the mitochondria increases its activity, enhancing ATP production through the glycolysis pathway (94–97). Fourth, the binding of HK2 to VDAC improves the stabilization of the mitochondrial membrane, leading to decreased reactive oxygen species (ROS) generation (96, 97). Finally, the binding of HK2/VDAC prevents Bax/Bak unbinding from the mitochondria and apoptosis (97, 98). Therefore, HK2 seems to be important for sustained cancer growth and has been suggested to be a marker of progression and tumor aggressiveness (91–103).

The earliest studies documenting the relationship between HK activity and thyroid carcinogenesis date back to the 1980s (99, 103). Rijksen et al. (103) showed no differences in HK biochemical characteristics when comparing MTC and ATC. Only the affinity of HK for its substrate was higher in ATC than in MTC. Nahm et al. (89) studied 342 PTC samples and found higher HK2 levels in 50% of PTC samples harboring the BRAF^V600E^ mutation. Recently, we demonstrated that HK activity is higher in the BCPAP and TPC1 cell lines than in non-tumor cells (88). Interestingly, HK activity in the cytosolic and mitochondrial fractions was significantly different between the two thyroid cancer cell lines. TPC1 cells that have RET/PTC translocation, showed equally distributed HK activity in the two subcellular fractions, while BCPAP (BRAF mutated) cells had higher HK activity in the mitochondrial fraction (88). According to Hooft et al. (100, 101), HK expression is similar between metastatic and primary DTC tumors, and positive ^18^FDG uptake on PET is associated with higher HK1 expression, however, mitochondria-bound HK was not evaluated in this study.

### Pyruvate Kinase

The PK enzyme catalyzes the last reaction of the glycolytic pathway. It is responsible for the conversion of phosphoenolpyruvate and ADP into pyruvate and ATP, respectively. PK monomer is composed of one active site, three main domains (denominated A, B and C), and a small N-terminal domain (104, 105). The C domain is the dimerization interface of the enzyme, and enzyme dimers can interact in a dimer–dimer configuration forming a tetrameric protein (104).

The PK isoform M1 (PKM1) is a constitutive tetramer exhibiting the highest activity that is expressed in tissues with high metabolic demand, such as brain, heart, and skeletal muscle (106, 107). PK isoform M2 (PKM2) is found in normal proliferating cells, but it is predominantly expressed in tumor cells and seems to be important for cancer cell metabolic adaptation (104, 108–110). The PKM2 isoform, in contrast to PKM1, can occur as dimers or tetramers, depending on the presence or absence of allosteric regulators (104, 105). The main positive allosteric regulator of PKM2 is fructose-1,6-biphosphate (Fructose-1,6-P2) that stabilizes the active tetrameric form of the enzyme (104, 105). PKM2 activity is negatively regulated by acetylation, phosphorylation, and oxidation. The phosphorylation of PKM2 at tyrosine 105 interferes with fructose-1,6-P2 binding and induces transformation from tetrameric to dimeric state. Also, the acetylation of PKM2 at lysine 305, or its oxidation at cysteine 358 decreases PKM2 activity (104, 105, 110). Decreased PKM2 activity leads to the accumulation of upstream glycolysis intermediates and consequently results in the deviation of metabolites to the PPP biosynthetic pathway and improved hexamine formation, nucleotide synthesis, and NADPH/NADP^+^ formation, contributing to the maintenance of redox homeostasis (110, 111). Therefore, when dimeric PKM2 is present in a tumor, less pyruvate is produced, limiting the mitochondrial substrate, what contributes to the metabolic shift from OXPHOS to aerobic glycolysis (104, 110).

Our group has shown that the human PTC cell lines, BCPAP and TPC1, express higher PKM2 mRNA levels compared to non-tumor cells, but no differences were found in PKM1 mRNA levels. However, the total activity of PK in PTC cells carrying the BRAF mutation (BCPAP) was higher than that in both non-tumor (NTHY-ori) and TPC1 (RET/PTC) cell lines, indicating that PKM2 enzymatic responses depend on the PTC driver mutation (88). It is believed that PKM isoform expression and activity change with tumor progression are linked to an increased tumor growth rate (112, 113). Feng et al. (113) showed that PKM2 expression in human PTC was associated with advanced tumor stages and lymph node metastasis. In addition, more intensive immunostaining of PKM2 was detected in PTCs harboring the BRAF mutation (113). Recently, Bikas et al. (114) showed that some thyroid cancer cells (FTC133 and BCPAP) characterized by glycolysis dependency overexpress PKM2. Although there are few studies in the literature, higher PKM2 expression in thyroid carcinomas appears to be significantly associated with the BRAF mutation, suggesting that this enzyme may be a potential therapeutic target in this type of cancer.

The relationship between dimeric and tetrameric PKM2 states has been described as a key factor for cell proliferation (111, 112, 115, 116). Several studies have shown that dimeric PKM2 can be translocated to the cell nucleus, where it directly interacts with multiple transcriptional factors and acts as a transcriptional coactivator involved in the upregulation of glycolytic genes, cell migration, and adhesion. The STAT3 signaling pathway seems to be involved in these effects of PKM2, which could be responsible for metastatic progression (112, 115, 117–119).

### Lactate Dehydrogenase (LDH)

Lactate production plays a critical role in tumor biology. Due to their high glucose consumption, cancer cells display increased lactate production regardless of oxygen availability (31–36). Lactate is formed by the conversion of pyruvate and NADH in a reversible reaction catalyzed by lactate dehydrogenase (LDH). Although the isoforms of LDH are expressed in several tissues, LDHA is upregulated in a wide range of tumor tissues (120, 121). The LDHA converts pyruvate into lactate preferentially, while the lactate dehydrogenase B (LDHB) acts in opposite way (120). When PKM2 activity decreases, a change in the cytosolic NAD^+^/NADH ratio occurs (104), what negatively impacts on the pyruvate to lactate conversion by LDHA. The downregulation of LDHA produces energy imbalance and oxidative stress leading to cell death (121, 122). Mirebeau-Prunier et al. (123) showed a lower LDHA/LDHB ratio in thyroid oncocytomas and follicular thyroid tumors. The downregulation of LDHA expression is related to the upregulation of estrogen-related receptor alpha, leading to changes in the oxidative metabolic profile of the tumor (123). In contrast, Kachel et al. (124) showed that LDHA is overexpressed in FTC and PTC compared to non-tumor tissues and its levels were even higher in UTC, suggesting that LDHA could be used as a biomarker of tumor aggressiveness. Comparing two PTC cell lineages, BCPAP and TPC1, we did not find differences in LDHA mRNA expression when compared to non-tumor cells. However, both tumor cell lineages had higher LDH activity and lactate production rates (88).

### Monocarboxylate Transporter (MCT)

New evidence has identified MCT as an essential factor in thyroid cancer phenotype (38). MCTs are part of a family of transporters with more than 14 defined members. In the thyroid, MCT10 and MCT8 have been characterized. However, only MCTs 1–4 act in the transport of monocarboxylates, such as lactate, pyruvate, and ketone bodies (38). MCT isoform 1 (MCT1) is a bidirectional lactate transporter present in the plasma membrane that mediates the influx of lactate into the cell. MCT1 is also found in the outer mitochondrial membrane, transporting lactate from the cytosol to the mitochondrial matrix, which increases ATP production *via* OXPHOS (38). MCT isoform 4 (MCT4) is a low-affinity lactate transporter that mediates lactate efflux from cells. In tumor cells, these transporters are important for the maintenance of glycolysis under hypoxic conditions or in normoxia so that tumor cells can utilize lactate and other high-energy substrates produced (38, 121, 124). The lower lactate levels found in the media of some cancer cells in culture suggest a higher lactate uptake *via* MCTs, allowing them to generate large amounts of ATP *via* OXPHOS (121, 124, 125). On the other hand, higher lactate levels outside of cells indicate that MCT4 is responsible for the export of lactate in some cancer cells (38, 125). Therefore, according to Curry et al. (125), MCT1 could be used as an indicator of higher OXPHOS, and MCT4 can be used as a marker of glycolytic metabolism. In head and neck cancers, the expression of MCT4 has been associated with a higher tumor stage and poorer clinical outcomes (125). Curry et al. (125) also described crosstalk between PTC thyrocytes and adjacent fibroblasts with a glycolytic phenotype, resulting in the production of high amounts of lactate, which is transported outside the cell by MCT4. On the other hand, PTC cells showed greater MCT1 staining, which allows lactate intake and consumption by mitochondrial oxidation. From these adjustments, PTC cells may obtain the energy to survive, proliferate, and metastasize (126).

## Role of ROS in Thyroid Cancer Metabolism

During the process of tumor progression, some metabolic changes are associated with high levels of ROS (126, 127). High levels of ROS can generate oxidative stress due to an imbalance between ROS production and antioxidant defenses. The major source of ROS seems to be the mitochondria where they are produced as a consequence of OXPHOS. Therefore, decreased mitochondrial metabolism may be important for decreasing ROS production and protecting cancer cells from death. However, improved antioxidant defenses have been observed as a compensatory mechanism in response to ROS generation, which is often increased in several tumors, including thyroid carcinomas (126, 127).

The NADPH oxidase/dual oxidase enzymes, also called NOXs and DUOXs, are specialized sources of ROS that are widely expressed in a variety of tissues, including the thyroid gland (128, 129). In the thyroid, DUOX1 and DUOX2 are the main producers of H_2_O_2_, although thyrocytes also express NADPH oxidase 4 (NOX4) that is prominently expressed in PTC and corresponds to an important source of ROS (128–130). In fact, there is a significant positive association between BRAF oncogene activation and NOX4 expression (130).

The oncocytic tumor cells are characterized by the presence of a high number of mitochondria probably due to an imbalance between mitochondria biogenesis and destruction; these cells depend on OXPHOS for energy conservation and produce high ROS levels (131). According to Maximo and Sobrinho-Simoes (132), the increased ROS production of oncocytic cells could be secondary to the decreased activities of complexes I and III of the electron transport chain (133). In fact, the oncocytic phenotype is associated with disruptive mutations in complex I subunits genes encoded by mitochondrial DNA (134), which might be involved in tumor cell death due to inefficient metabolic adaptation, since the induction of the Warburg phenotype through the stabilization of HIF-1 alpha depends on normal complex I function that sustains tumor growth (135).

Interestingly, Paik et al. (136) showed that ROS can also increase glucose metabolism through HIF-1 activation. Using endothelial cells, they showed that increased ROS levels are accompanied by higher glucose uptake and lactate production when these cells are subjected to hypoxia. However, the glycolytic phenotype is blocked by HIF-1 stabilization, suggesting that ROS-driven HIF-1α accumulation accelerates glycolysis in endothelial cells (136).

Reactive oxygen species also change cell metabolism through AMP kinase (AMPK) protein activation (137). Intracellular ROS can stimulate AMPK, a metabolic stress-sensing cytosolic enzyme that regulates energy consumption and production processes (137). Although controversy remains in the literature relative to AMPK pathway involvement in tumorigenesis and cancer progression, several studies have demonstrated that activated AMPK causes cell-cycle arrest and has a strong antiproliferative effect in different cancer cell lines (137–139). AMPK activation also increases GLUT-1 protein expression, glucose uptake, and utilization of the glycolytic pathway in both non-tumor models and in tumor models, including PTC models (137–139). Some studies have shown that disruption of AMPK activity induces the Warburg effect in tumor cells (137, 140, 141). In an AMPK-deficient mouse model of Peutz–Jeghers syndrome, mammalian target of rapamycin protein (mTOR) is upregulated and HIF-1 promotes higher HK2 and Glut1 expression and increased glucose utilization by tumors (142). According to Bikas et al. (114), AMPK activation seems to be involved in the glucose metabolism dependence that is observed in some PTC cells. We described greater expression of the active phosphorylated form of AMPK in PTC tissue samples and in PTC tumor cell lineages in culture compared to that in non-tumor tissues (139, 143). However, new studies are necessary to understand the role of AMPK in human thyroid cancer, especially in terms of metabolic control, cell growth, apoptosis, and survival.

## Targeting Metabolism in Thyroid Cancer

Considering the diversity of thyroid tumors and their distinct metabolic requirements, establishing a unique strategy for cancer therapy is not an easy task. However, some drugs for cancer therapy that target the tyrosine kinase receptors signaling cascade are currently being used. These proteins constitute a group of enzymes involved in the control of mitogen signals, energy status, cell survival, and angiogenesis (144, 145). Several tyrosine kinase inhibitors (TKIs) have been developed for thyroid carcinoma treatment, but not all of them have received approval from international health agencies (145). Many TKIs are still in the initial clinical phase of study. Sorafenib, lenvatinib, vandetanib, and cabozantinib are multikinase inhibitors approved by the Food and Drug Administration and the European Medical Agency (EMA) for use in patients with advanced thyroid carcinomas. In fact, these patients had significantly increased progression-free survival rates with the use of these agents (145).

**Figure 4:**
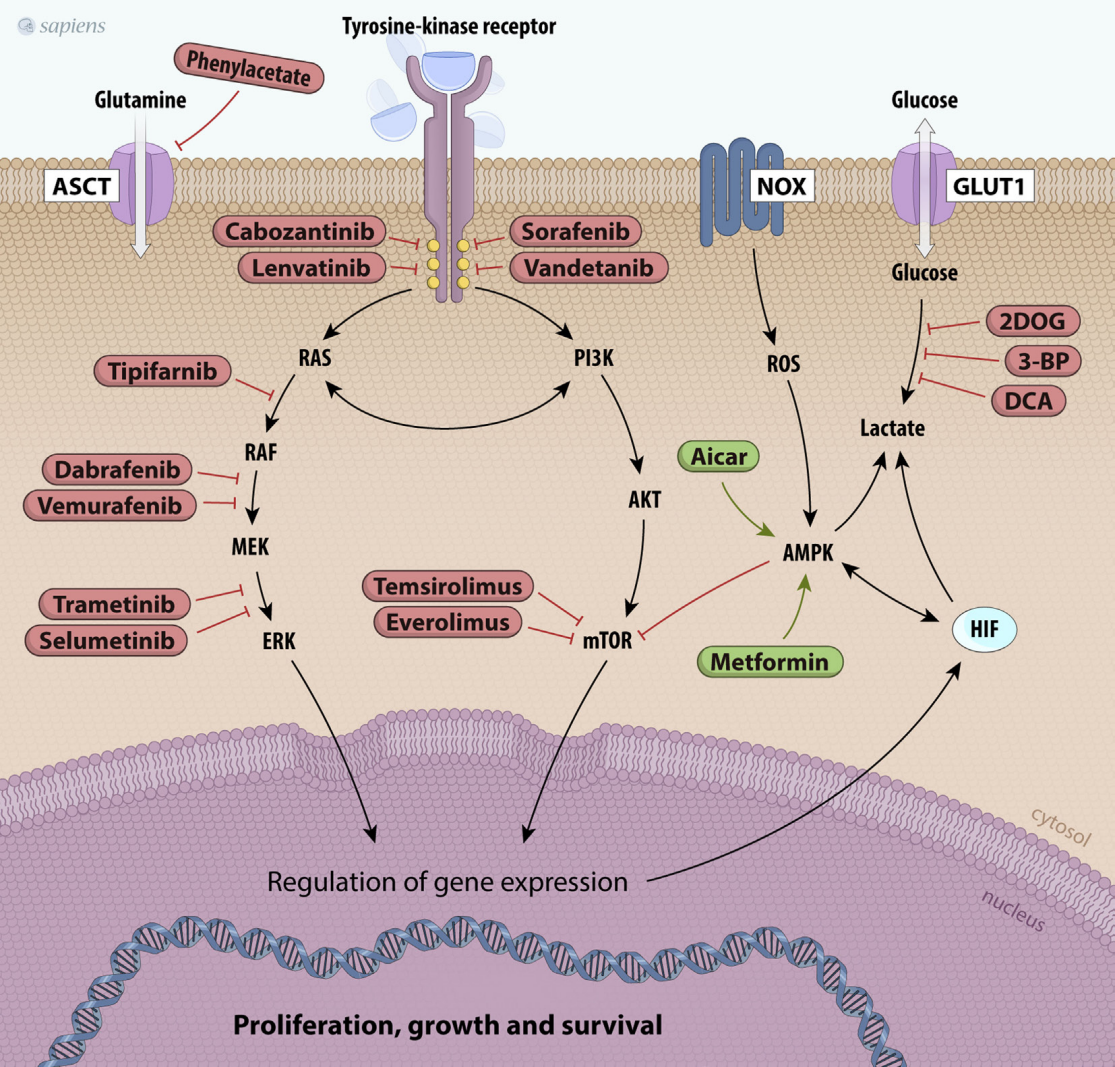
Scheme of principal potential therapeutic targets. The products of mutated genes activated in cancer cells are targets for anticancer drug therapy [Valerio et al. (144), Bible and Ryder (147), and De Falco et al. (148)]. Several kinase inhibitors along the RET/papillary thyroid carcinoma, RAS, PI3K/AKT/mTOR, and MAPK signaling pathways are shown in red. Metabolic alterations in thyroid cancer cells can also be inhibited, such as glutamine uptake (phenylacetate) and glucose metabolism (2DOG, 3-BP, DCA). Finally, other drugs can activate molecules like AMPK, such as metformin and AICAR, which are shown in green. Abbreviations: 2DOG, 2-deoxyglucose; 3-BP, 3-bromopyruvate; DCA, dichloroacetate, AICAR, 5-aminoimidazole-4-carboxamide-ribonucleoside, NOX, NADPH oxidade; MAPK, mitogen-activated protein kinase; AMPK, AMP kinase.

In addition to TKIs, other downstream targets can regulate metabolic pathways (Figure 4). PI3K was described as a component of the insulin receptor intracellular signaling pathway and its main substrate is AKT. Once activated, AKT increases phosphorylation events in both the cytoplasm and the nucleus, stimulating glucose uptake; glycolytic flux; inhibition of apoptosis; and activation of mTOR, an important regulator of metabolism and cancer growth (145, 146). PI3K/AKT signaling is inhibited by the tumor suppressor phosphatase and tensin homolog (PTEN), which is often mutated in many types of tumors. Therefore, the loss of PTEN activity allows PI3K/AKT constitutive activation (145, 146). LY294002 is a classic molecule that inhibits PI3K and has already been tested in some carcinomas, including thyroid cancer. Currently, other new drugs targeting PI3K/AKT have been tested in several carcinomas and are in different clinical trial phases of study (144, 145).

Mammalian target of rapamycin protein is the main downstream effector of PI3K/AKT, and its upregulation is involved in tumorigenesis (144–146). Our group demonstrated that the activation of PI3K/AKT/mTOR inhibits iodide uptake by diminishing sodium/iodide symporter transporter (NIS) expression in non-tumor cells (149). Loss of iodide uptake is a negative process in the course of disease evolution, since thyroid cancer treatment depends on the administration of radioactive iodine (145). Several mTOR inhibitors (rapamycin analog), such as everolimus, have been studied in preclinical and clinical trials, including thyroid carcinomas (144, 145). Recently, phase II studies using everolimus in patients with advanced thyroid cancer have reported a partial response and sustained stable disease in a small proportion of patients (5–45%, respectively), with progression-free survival rates of approximately 11–16 months (150). The use of everolimus as monotherapy shows moderate effects, but its clinical relevance mainly derives from its relatively low toxicity profile (144, 151). The combination of another mTOR inhibitor (temsirolimus) and other drugs (MEK inhibitors) has shown synergic effects *in vitro* and is being tested in clinical trials (152, 153).

AMP kinase is a potent physiological mTOR inhibitor. Under low ATP/AMP ratio conditions, AMPK is activated, leading to metabolic adaptations, such as increased catabolism and decreased anabolism that are partially mediated by mTOR inhibition. The crosstalk between mTOR and AMPK has been extensively studied (137, 138, 140). The first evidence associating AMPK with cancer development was the discovery of liver kinase B1 (LKB1). LKB1 is a serine/threonine kinase and the major upstream kinase responsible for AMPK activation through phosphorylation (138). LKB1 is recognized as a tumor suppressor that associates bioenergetics with cell growth control and downregulation of mTOR activity through AMPK activation (138, 142). We have studied the effects of a pharmacological AMPK activator (AICAR) on PTC cell lines and observed decreased cell proliferation and the induction of apoptosis (139). These results suggest that AMPK may be a good target for thyroid cancer therapy (Figure 4). Epidemiological studies reported that in thyroid cancer patients who are also diabetic, metformin, an oral anti-diabetic drug, can activate AMPK, resulting in a reduced tumor size and higher remission rates (154). Interestingly, our group showed that the expression and activity of AMPK are increased in human PTC and in PTC cell lines (BCPAP and TPC1) compared to those in non-tumor tissues and non-tumor cell lines (139, 143). As PTCs are well-differentiated and slow-growing carcinomas, it is believed that increased AMPK activation could impair tumor growth.

Although tumor energy metabolism has common characteristics, most molecular targets that may be used for tumor treatment are ubiquitously expressed and function in the entire body. Therefore, it is difficult to produce specific effects only in tumor cells. Glycolytic inhibitors, such as 2-deoxyglucose (2DOG) and 3-bromopyruvate (3-BP), can be used as adjuvant agents to sensitize tumors. Unlike 2DOG, 3-BP acts in many targets and inhibits HK, GAPDH, and MCT activities, thus leading to decreased aerobic glycolysis (155). Furthermore, inhibition of PFK1 activity, a rate-limiting step of glycolysis, is also an interesting strategy in cancer therapy. This protein is activated by fructose-2,6-bisphosphate (F2,6BP), which is produced by PFKFBs. A small molecule, 3-(3-pyridinyl)-1-(4-pyridinyl)-2-propen-1-one, has been found to inhibit PFKFB3, leading to decreased glycolytic flux and slower tumor growth (Figure 4) (156).

Other attractive targets for cancer therapy include the inhibition of lactate production. Some previous studies have shown that loss of LDHA function by dichloroacetate (DCA) results in dramatically diminished cellular transformation or xenograft tumor growth in breast cancer (157). Glutaminolysis and amino acid metabolism are very important for tumors. Glutamine is the most abundant amino acid in the plasma and is heavily consumed by tumor cells. Therapies that decrease plasma glutamine concentrations induce tumor regression and prevent muscular catabolism, an endogenous source of glutamine. Phenyl acetate is a promising drug that can reduce the availability of glutamine in the blood and shows low toxicity (158).

Finally, another strategy targeting metabolism in tumors is diet restriction, fasting, or a ketogenic diet (a low-carbohydrate and high-fat diet) (159–162). The hypothesis is based on the glucose dependency of many tumor types. Interestingly, these diets do not increase plasma glucose levels, but produce ketone bodies that can be used as a carbon source for energy production in oxidative processes, altering the Warburg phenotype (159–162).

## Conclusion

In summary, although thyroid cancer studies are emerging, the mechanism of tumor progression remains unclear. As described previously, metabolic reprogramming is the hallmark of cancer cells. Recent molecular studies in thyroid cancer revealed that oncogenes and tumor suppressor genes not only control growth and apoptotic phenotypes of thyroid carcinomas, but also directly affect cellular energy metabolism and are implicated in the Warburg phenotype. The higher glucose and glutamine consumption associated with the disruption of mitochondrial OXPHOS create a favorable environment for tumor progression.

## Author Contributions

RC—writing and elaborating the figures. RF—writing and reviewing the manuscript. DC—writing and reviewing the final version.

## Conflict of Interest Statement

The authors declare that the research was conducted in the absence of any commercial or financial relationships that could be construed as a potential conflict of interest.
